# Investigation of Integron-Associated Resistance Gene Cassettes in Urinary Isolates of *Klebsiella pneumoniae* in Yasuj, Southwestern Iran During 2015–16

**Published:** 2020

**Authors:** Fariba Jahanbin, Masoud Marashifard, Sanaz Jamshidi, Maryam Zamanzadeh, Masumeh Dehshiri, Seyed Ali Asghar Malek Hosseini, Seyed Sajjad Khoramrooz

**Affiliations:** 1. Department of Basic Sciences, Islamic Azad University, Yasuj Branch, Yasuj, Iran; 2. Treatment Management of Social Security Organization of Kohgiluyeh and Boyer-Ahmad Province, Yasuj, Iran; 3. Cellular and Molecular Research Center, Yasuj University of Medical Sciences, Yasuj, Iran; 4. Student Research Committee, Yasuj University of Medical Sciences, Yasuj, Iran; 5. Medicinal Plants Research Center, Yasuj University of Medical Sciences, Yasuj, Iran; 6. Department of Microbiology, Faculty of Medicine, Yasuj University of Medical Sciences, Yasuj, Iran

**Keywords:** Antibiotic resistance, Integrons, Iran, *Klebsiella pneumoniae*

## Abstract

**Background:**

Growing antibiotic resistance among urinary opportunistic pathogens such as *Klebsiella pneumoniae (K. pneumonia)* has created a worrisome condition in the treatment of the Urinary Tract Infections (UTIs) in recent years. Integrons play a significant role in the dissemination of antibiotic resistance genes. The present study was conducted to investigate class 1–3 integrons and the corresponding resistance gene cassettes in urinary *K. pneumoniae* isolates.

**Methods:**

In this study, from December 2015 to September 2016, a total of 196 *K. pneumoniae* isolates were collected from the patients with UTI referred to medical diagnostic laboratories in Yasouj, Southwestern Iran. Antibiotic susceptibility patterns of isolates were determined using 12 antibiotics by the disc diffusion method. Polymerase Chain Reaction (PCR) was used for detection of integron genes (*intI1, intI2, and intI3*). The variable regions of integrons were amplified by PCR and sequenced to identify the corresponding gene cassettes.

**Results:**

Thirty-nine different antibiotic resistance profiles were observed among *K. pneumoniae* isolates. Only 12.2% of *K. pneumoniae* isolates were found to harbor the *intI1* gene. While 17 (60.7%) out of 28 Multidrug Resistance (MDR) *K. pneumoniae* isolates carried the *intI1* gene, only 4.2% of non-MDR isolates harbored *intI1* gene. Totally 7 different gene cassette arrays were found in the *intI1* gene of *K. pneumoniae* isolates. The aadA1 was the most prominent gene cassette. Also, high frequency of *dfrA* containing gene cassettes was observed.

**Conclusion:**

Continuous monitoring and characterization of integrons and their associated gene cassettes could be helpful in controlling the rising rate of antibiotic resistance.

## Introduction

Urinary Tract Infection (UTI) is one of the most prominent infectious diseases in both community and healthcare setting. *Escherichia coli (E. coli*) is considered as the most prominent uropathogen which accounts for 75–90% of all UTIs in both inpatients and outpatients followed by *Klebsiella pneumoniae (K. pneumonia)* 1,2. The increasing resistance rate to antibiotics among UTI causing organisms makes the empiric treatment of these infections very challenging 3. Increasing antibiotic usage, as well as horizontal transfer of antibiotic resistance genes located on various types of mobile DNA elements as plasmids, transposons and gene cassettes in integron, has facilitated the development of Multidrug Resistance (MDR) in *Enterobacteriaceae* family 4. Integrons are genetic elements that play a significant role in the transmission of multidrug resistance genes in several gram-negative bacteria 5. An integron is structurally composed of three genetic elements; *integrase* gene (*intI*) which is responsible for site specific recombination of mobile gene cassettes, attachment site (*attI*) and the promoter (Pc) 6. Gene cassettes are discrete genetic elements consisting of the single Open Reading Frame (ORF) and a recombination site (a 59 *bp* element) known as the *attC* site 7. The integrase mediates the integration of circular gene cassettes between *attI* and *attC* sites 8. Integrons are classified into several classes, based on the amino acid sequence homology of *intI* gene, among them class 1, 2 and 3 are usually recovered from clinical isolates 9,10. Class1 integron has been reported in different studies as the most frequent class identified in clinical isolates 9. Class1 integron contains variable regions of gene cassettes that are sometimes absent in the structure of integron, flanked by two highly conserved regions; 5′-conserved segment (5′-CS) and 3′-conserved segment (3′-CS) 6. Gene cassettes are promotorless variable regions of integrons, which encode antibiotic resistance phenotype, located between *attC* and *attI* region. Their expression depends on the integron promoter (Pc) which relies on the 5′-CS in the case of class1 integron. Different arrays of cassettes have been reported and most arrays had two or three gene cassettes 11.

At least 130 different gene cassettes including diverse resistance genes have been identified, mainly conferring resistance to different classes of antibiotics including aminoglycosides, β-lactams, chloramphenicol, trimethoprim, erythromycin, and rifampicin 11,12. The most prominent gene cassettes within class 1 integron among *Enterobacteriaceae* family are *aadA* and *dfrA*, which confer resistance to streptomycin and trimethoprim, respectively 13. Although some studies have reported the prevalence of class 1–3 integrons and gene cassettes in *E. coli*, *Pseudomonas aeruginosa* and *Acinetobacter baumannii* isolates in Iran 14–17, there are limited data regarding the distribution of gene cassettes in *K. pneumoniae* isolates from Iran 18. Therefore, in the present study, an attempt was made to investigate the gene cassettes in addition to the prevalence of class 1–3 integrons and antibiotic resistance patterns in a series of clinical isolates of *K. pneumoniae* in Yasuj, Southwestern Iran.

## Materials and Methods

### Bacterial isolation

From December 2015 to September 2016, 196 non-repetitive isolates of *K. pneumoniae* were collected from the urine samples of patients with UTI who were referred to medical diagnostic laboratories in Yasuj, Southwestern Iran. Each urine sample was cultured on EMB and Blood Agar (Merck, Germany) and incubated at 37*°C* for 24 *hr*. Using conventional biochemical tests on culture media such as Methyl red-Voges Proskauer (MR-VP), Triple Suger İron (TSI), Sulfide İndole Motility (SIM), Simmons Citrate, and Urea Agar (Merck, Germany), identification of *K. pneumoniae* isolates was performed 19. Verified isolates of *K. pneumoniae* were stored at −20*°C* in TSB medium (Merck, Germany) with 15% glycerol for next steps. This study was approved by the ethical committee of Yasuj University of Medical Sciences.

### Antibiotic susceptibility testing (AST)

Susceptibility of *K. pneumoniae* isolates was determined by the disk diffusion method according to the Clinical and Laboratory Standards Institute (CLSI) guidelines 20. Twelve antibiotic disks (MAST, UK) including amoxicillin/clavulanic acid (AMC; 30 *μg*), cephalothin (CEP; 30 *μg*), ceftazidime (CAZ; 30 *μg*), imipenem (IMP; 10 *μg*), chloramphenicol (CLR; 30 *μg*), nitrofurantoin (NI; 300 *μg*), tetracycline (TET; 30 *μg*), gentamycin (GEN; 10 *μg*), amikacin (AMI; 30 *μg*), ciprofloxacin (CIPR; 5 *μg*), nalidixic acid (NAL; 30 *μg*), and trimethoprim-sulfamethoxazole (SXT; 25 *mg*) were used. *K. pneumoniae* ATCC 700603 was used as the control for antibiotic resistance. MDR was outlined as acquired non-susceptibility to a minimum of one agent in 3 or more antimicrobial classes 21.

## DNA extraction

The boiling method with some modification was used to extract the genome of bacteria 22. In short, 0.5 McFarland bacterial suspension was prepared first, and then 300 *μl* of it was transferred to the 1.5 *ml* microtube containing sterile distilled water. The suspension within the microtube was homogenized with the vortex and then boiled for 10 *min* at 100*°C* in a water bath. In the next step, microtube was centrifuged for 10 *min* at 14000 *g* and the supernatant containing genome was transferred to the 0.5 *ml* microtube and kept at 18*°C* until the next step.

### PCR for detection of integron genes

Polymerase Chain Reaction (PCR) was carried out for detection of *integron genes (intI1, intI2, and intI3)* using the primers described in table 1 23,24. Each reaction mixture in a final volume of 25 *μl* contained 12.5 *μl* of 2X Master mix (Amplicon, Denmark), 0.3 *μl* of each primer (20 *pM/μl*), 2.5 *μl* of DNA template and 9.4 *μl* of distilled water.

**Table 1.** Primers used for the detection of class 1–3 integrons and variable region of class 1 integrons

**Target genes**	**Oligonucleotide sequences of primers (5′ to 3′)**	**Expected size (*bp*)**	**Reference**
***intI1***	F: CCTCCCGCACGATGATC R: TCCACGCATCGTCAGGC	280	Kraft *et al* (1986)
***intI2***	F: TTATTGCTGGGATTAGGC R: ACGGCTACCCTCTGTTATC	233	Goldstein *et al* (2001)
***intI3***	F: AGTGGGTGGCGAATGAGTG R: TGTTCTTGTATCGGCAGGTG	600	Goldstein *et al* (2001)
**Variable region of class 1 integrons**	5′-CS: GGCATCCAAGCAGCAAG 3′-CS: AAGCAGACTTGACCTGA	variable	Levesque *et al* (1995)

The planned conditions in the thermocycler (Bio-Rad, T100, USA) for amplification of each of the genes examined were as follows; initial denaturation at 94*°C* for 4 *min*; 35 thermal cycles of denaturation at 94*°C* for 40 *s*, annealing at 58*°C* (For *intI1* genes) or 51*°C* (For *intI2* and *intI3* genes) for 30 *s*, and extension at 72*°C* for 40 *s*; and a post-PCR final incubation at 72*°C* for five minutes. The amplified PCR products were electrophoresed on 1.5% agarose gel containing 0.5 *μg/ml* ethidium bromide and visualized under a UV transilluminator (Major science, Taiwan).

### Characterization of gene cassettes inserted in the variable regions of class 1 integrons

PCR was carried out for amplification of variable regions of class 1 integron similar to the planned conditions for the detection of *intI1* gene, except that the annealing temperature was 60*°C* using primers described in table 1 25. For each integron-positive isolate, some of the PCR product was electrophoresed and, after ensuring that the expected fragment was present, the remaining PCR product was sent to Macrogen company (Seoul, South Korea) for sequencing.

Obtained sequences were submitted in the NCBI database and blasted by online BLAST search (http://www.ncbi.nlm.nih.gov/BLAST/).

### Statistical analysis

Data were analyzed with SPSS software (Version 15, Chicago, IL, USA). The chi-square and Fisher’s exact tests were used for determining the association between presence of *intI* genes and antibiotic resistance status. A p<0.05 was regarded statistically significant.

## Results

### Antibiotic susceptibility test results

Among 196 studied isolates of *K. pneumoniae*, 137 isolates (69.9%) were collected from females and the remaining 59 isolates (30.1%) were obtained from male patients. The results of the AST showed that resistance to amoxicillin is highest (38.8%). Resistance rate to other antibiotics was as follows; they were cephalothin (32.1%), nitrofurantoin (22.4%), ceftazidime (12/8%) trimethoprim-sulfamethoxazole (9.7%), tetracycline (8.7%), gentamycin (5.1%), nalidixic acid (3.6%), chloramphenicol (3.6%), ciprofloxacin (2.6%), and amikacin (1%). There was not any imipenem-resistant isolate. Twenty-eight (14.3%) isolates were MDR and 39 different antibiotic resistance profiles were observed (Table 2).

**Table 2.** *intI1* gene presence status in *K. pneumoniae* isolates according to antibiotic susceptibility pattern

**Antibiotics**	***intI1* gene presence**	**Antibiotic resistance pattern**			**p-value**

		**Susceptible (%)**	**Intermediate (%)**	**Resistant (%)**	
**SXT**					
	*intI1*+ (24)	7 (29.2)	0	17 (70.8)	<0.001
	*intI1*− (172)	169 (98.3)	1 (0.6)	2 (1.2)	
**TET**					
	*intI1*+ (24)	7 (29.2)	6 (25)	11 (45.8)	<0.001
	*intI1*− (172)	162 (94.2)	4 (2.3)	6 (3.5)	
**NI**					
	*intI1*+ (24)	9 (37.5)	8 (33.3)	7 (29.2)	0.642
	*intI1*− (172)	79 (45.9)	56 (32.6)	37 (21.5)	
**GEN**					
	*intI1*+ (24)	19 (79.2)	1 (0.5)	4 (16.7)	0.003
	*intI1*− (172)	166 (96.5)	0	6 (3.5)	
**AMI**					
	*intI1*+ (24)	24 (100)	0	0	1
	*intI1*− (172)	168 (97.7)	2 (1.2)	2 (1.2)	
**CIP**					
	*intI1*+ (24)	17 (70.8)	3 (12.5)	4 (16.7)	<0.001
	*intI1*− (172)	169 (98.3)	2 (1.2)	1 (0.6)	
**NAL**					
	*intI1*+ (24)	18 (75)	2 (8.3)	4 (16.7)	<0.001
	*intI1*− (172)	168 (97.7)	1 (0.6)	3 (1.7)	
**CLR**					
	*intI1*+ (24)	17 (70.8)	2 (8.3)	5 (20.8)	<0.001
	*intI1*− (172)	169 (98.3)	1 (0.6)	2 (1.2)	
**AMC**					
	*intI1*+ (24)	1 (4.2)	2 (8.3)	21 (87.5)	<0.001
	*intI1*− (172)	50 (29.1)	67 (39)	55 (32)	
**CEP**					
	*intI1*+ (24)	6 (25)	2 (8.3)	16 (66.7)	0.001
	*intI1*− (172)	100 (58.1)	25 (14.5)	47 (27.3)	
**CAZ**					
	*intI1*+ (24)	11 (45.8)	4 (16.7)	9 (37.5)	<0.001
	*intI1*− (172)	154 (89.5)	2 (1.2)	16 (9.3)	

SXT=Trimethoprim-Sulfamethoxazole, TET=Tetracycline, NI=Nitrofurantoin, GEN=Gentamycin, AMI=Amikacin, CIP=Ciprofloxacin, NAL=Nalidixic acid, CLR=Chloramphenicol, AMC=Amoxicillin/Clavulanic acid, CEP=Cephalothin, CAZ=Ceftazidime.

### Detection of integrons and characterization of gene cassettes

Among the 196 *K. pneumoniae* isolates, class 1 integron (*intI1*) was identified in 24 (12.2%) isolates while *intI2* and *intI3* genes were not found in any of the studied *K. pneumoniae* isolates. In table 2, the frequency of *intI1* gene-positive isolates in each of the resistance profiles is shown.

Significant differences were observed between susceptibility patterns of trimethoprim-sulfamethoxazole, tetracycline, amoxicillin/clavulanic acid, ceftazidime, ciprofloxacin, chloramphenicol, nalidixic acid, cephalothin, gentamycin and presence of class1 integron in *K. pneumonia* isolates (Table 3). While 17 out of 28 (60.7%) MDR *K. pneumoniae* isolates harbored the *intI1* gene, only 4.2% of non-MDR isolates carried *intI1* gene (p<0.001).

**Table 3.** Frequency of *intI1* gene-positive isolates in each of the resistance profiles

**No.**	**Resistance phenotypes**	**No. of isolates**	**No. of** ***intI1*** **gene-positive isolates**
**1**	No resistance (0)	68	0
**2**	SXT(1)	1	1
**3**	AMC(1)	20	1
**4**	CEP(1)	12	0
**5**	CAZ(1)	3	0
**6**	NI(1)	25	0
**7**	TET(1)	4	2
**8**	AMI(1)	1	0
**9**	SXT, AMC(2)	2	2
**10**	AMC, CEP(2)	15	0
**11**	AMC, CAZ(2)	1	1
**12**	AMC, NI(2)	5	0
**13**	CEP, CAZ(2)	2	0
**14**	CEP, NI(2)	3	0
**15**	CAZ, NI(2)	1	0
**16**	SXT, AMC, CEP(3)	2	2
**17**	SXT, AMC, CLR(3)	2	1
**18**	AMC, CEP, TET(3)	1	1
**19**	AMC, CEP, GEN(3)	1	0
**20**	CAZ, AMC, CEP(3) (non MDR)	5	0
**21**	NAL, AMC, CEP, TET(4)	1	0
**22**	SXT, AMC, CEP, CAZ(4)	1	1
**23**	SXT, AMC, CEP, GEN(4)	2	2
**24**	AMC, CEP, CAZ, NI(4)	1	0
**25**	AMC, CEP, CAZ, GEN(4)	1	0
**26**	AMC, CEP, NI, TET(4)	1	1
**27**	AMC, CEP, TET, GEN(4)	1	0
**28**	CIPR, NAL, AMC, CEP, GEN(5)	1	0
**29**	NAL, AMC, CEP, CAZ, NI(5)	1	0
**30**	SXT, AMC, CEP, CAZ, TET(5)	1	1
**31**	AMC, CEP, CAZ, NI, TET(5)	2	1
**32**	AMC, CEP, CAZ, GEN, AMI(5)	1	0
**33**	SXT, AMC, CEP, CAZ, CLR, TET(6)	1	1
**34**	SXT, AMC, CEP, CAZ, NI, GEN(6)	1	1
**35**	SXT, AMC, CEP, CLR, TET, GEN(6)	1	0
**36**	CIPR, NAL, SXT, AMC, CEP, NI, TET(7)	2	2
**37**	SXT, AMC, CEP, CAZ, CLR, TET, GEN(7)	1	1
**38**	CIPR, NAL, SXT, AMC, CEP, CAZ, CLR, NI(8)	1	1
**39**	CIPR, NAL, SXT, AMC, CEP, CAZ, CLR, NI, TET(9)	1	1

**Figure 1.**
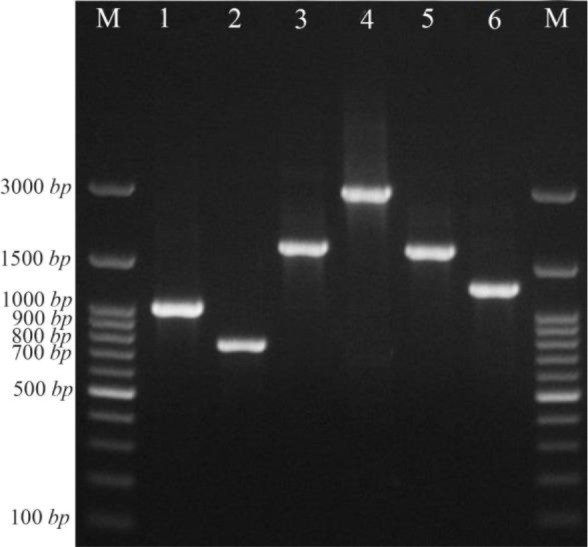
PCR assay for identification of some gene cassette arrays among *K. pneumoniae* isolates. Lane M: DNA marker, lane 1: *aadA1* (1000 *bp*), lane 2: *dfrA7* (750 *bp*), lane 3 and 5: *dfrA17-aadA5* (1650 *bp*), lane 4: *aadB-cat-blaOXA10-aadA1* (3000 *bp*), lane 6: *dfrA1-orfC* (1300 *bp*).

Eighteen out of 24 (75%) *intI1 positive K. pneumoniae* isolates were found to have 7 different gene cassette arrays including the *dfrA5, dfrA25, dfrA7, aadA1, dfrA17-aadA5, dfrA1-orfC* and *aadB-cat-bla*OXA10*-aad-A1* (Table 4, Figure 1). In the remaining 6 *intI1* positive *K. pneumoniae* isolates, variable region was not detected.

**Table 4.** Gene cassette arrays found in class 1 integrons and related resistance profiles

**Gene cassette arrays**	**Approximate amplicon size**	**No. of isolates (%)**	**Resistance profiles**
***dfrA5***	700 *bp*	1 (4.2)	AMC, CAZ
***dfrA25***	700 *bp*	1 (4.2)	AMC, CEP, TET
***dfrA7***	750 *bp*	1 (4.2)	CIPR, NAL, SXT, AMC, CEP, CAZ, CLR, NI, TET
***aadA1***	1000 *bp*	9 (37.5)	AMC TET (2 case) SXT, AMC SXT, AMC, CEP (2 case) AMC, CEP, NI, TET SXT, AMC, CEP, CAZ, NI, GEN CIPR, NAL, SXT, AMC, CEP, NI, TET
***dfrA1-orfC***	1300 *bp*	1 (4.2)	SXT, AMC, CLR
***dfrA17-aadA5***	1650 *bp*	4 (16.7)	SXT, AMC SXT, AMC, CEP, CAZ SXT, AMC, CEP, GEN CIPR, NAL, SXT, AMC, CEP, CAZ, CLR, NI, TET
***aadB-cat- blaOXA10-aadA1***	3000 *bp*	1 (4.2)	SXT, AMC, CEP, CAZ, TET

Among seven different cassette arrays found in *K. pneumoniae*, *aadA1* was the most frequent gene cassette detected in 9 (37.5%) out of 24 *intI1* positive isolates. *dfrA5*, *dfrA25*, *dfrA7*, *dfrA1-orfC* and *aadB-catbla*OXA10*-aadA1* gene cassette arrays were found in one isolate. Four isolates were found to be positive for *dfrA17-aadA5* gene cassette array.

## Discussion

Similar to previous reports from Iran and other countries, all of the studied *K. pneumoniae* isolates were found to be susceptible to imipenem 26–30. Also, in our previous study, only 1% of urinary *E. coli* isolates were imipenem resistant 22. The low and prudent administration of imipenem by physicians in Iran and consequently reducing selection pressure of this antibiotic on bacteria has made it the most reliable and most effective antibiotic against bacteria such as *E. coli* and *K. pneumoniae.* In the present study, the rate of resistance to trimethoprim-sulfamethoxazole (9.7%), tetracycline (8.7%), gentamycin (5.1%), nalidixic acid (3.6%), chloramphenicol (3.6%), ciprofloxacin (2.6%) and amikacin (1%) all were less than 10%. These relatively low rates were not observed in other studies from Iran and other countries in recent years. The resistance rate of urinary *K. pneumoniae* isolates in a study by Akram *et al* in India was reported as cotrimoxazole 53%, tetracycline 53%, gentamycin 53%, ciprofloxacin 47%, and amikacin 35% 31. Also, in another study in India by Mariya and Hatkar, the rate of resistance to co-trimoxazole and amikacin was 59.73% and 31.95%, respectively 29. In another study in Iran, the resistance rate of *K. pneumoniae* isolates was reported as co-trimoxazole 95.3%, tetracycline 64.7%, gentamicin 76%, chloramphenicol 57.3%, ciprofloxacin 43.3%, and amikacin 50.7% 32. Differences in the pattern of antibiotic resistance in different countries and even in different regions of a country may depend on different factors such as rate of access and use of different antibiotics, population, climatic conditions and public health levels 33.

In the present study, the highest rate of antibiotic resistance was observed for amoxicillin/clavulanic acid in 38.8% of *K. pneumoniae* isolates. Unfortunately, in Iran, the arbitrary use of this antibiotic among people has a very high prevalence that increases the selective pressure of the antibiotic on bacteria leading to resistance mechanisms.

In addition to amoxicillin/clavulanic acid, the resistance rate of our isolates was found to be higher than 10% for only three other antibiotics including cephalothin (32.1%), nitrofurantoin (22.4%) and ceftazidime (12/8%).

In the present study, class1 integron was detected in 12.2% of *K. pneumoniae*. This frequency is much lower than that reported by Ahangarzadeh Rezaee *et al* in northwest Iran (78%) 32. Considering that in the study of Ahangarzadeh Rezaee *et al*, 99.3% of isolates were MDR and in our study only 14.3% of isolates were MDR, this high difference between the presence of the integron in these two studies was not surprising. The above comparison may illustrate well the association between the emergence of MDR bacteria and the presence of integron. Also, Li *et al* and Lina *et al* reported a higher prevalence of class I integron compared to the current study 4,26. The prevalence of class I integron in their studies was 51.1% and 54%, respectively. On the other hand, in another study in Iran by Seyed Javadi *et al*, the prevalence of class I integrons among *K. pneumoniae* isolates (13.3%) was very close to our study 34.

Similar to other studies class 2 and class 3 integrons were not found in any of our *K. pnemoniae* isolates, while Ahangarzadeh Rezaee *et al* reported class 2 integrons in 13.4 % of their isolates 25,32,35,36.

Significant differences were observed between the presence of the *intI1* gene and susceptibility patterns of all antibiotics except amikacin and nitrofurantoin. Similar to our study, Li *et al* did not find a significant association between resistance to nitrofurantoin and the presence of class1 integron 4.

In the present study, 7 different cassette arrays were found in 75% of *intI1* positive *K. pneumoniae* isolates. Among them, *aadA1* was the most frequent gene cassette detected in 37.5% of 24 *intI1* positive isolates. The *aadA* type gene cassettes confer resistance to aminoglycosides such as streptomycin that was widely used for treating UTIs in the early years 37. Since streptomycin usage has been limited to some specific human diseases, its frequent application in agriculture and food animals, especially in livestock production, may bring development of resistance *via* acquiring resistance gene cassettes 37. Hence, this gene cassette can remain in animal pathogenic strains of *E. coli* and be disseminated to other strains by horizontal gene transfer mechanisms.

The high prevalence rate of gene cassette arrays with *dfr* genes in the current study, which codes for Dihydrofolate Reductase (DHFR) conferring resistance to trimethoprim, can reflect the wide use of trimethoprim in the treatment of urinary tract infections in recent years.

Similar to findings of previous studies 4,19, in the present work, 25% of *K. pneumoniae* isolates were found to carry class1 integron without gene cassettes. In a study from China performed on *K. pneumoniae* isolates, *dfrA1-orfC* was the most predominant cassette array among 10 different identified cassette arrays among which *dfrA5, aadA1* and *dfrA1-orfC* gene cassettes were also detected in our study 4. Also, Salimizand *et al* identified only 3 gene cassettes or cassette arrays in *K. pneumoniae* isolates including *arr-5, aacA4-orfD, and dfrA17-aadA5*, among which the latter cassette was found in our study 27.

In the present study, all isolates with the *dfrA7*, *dfrA17-aadA5,* and *dfrA1-orfC* gene cassette arrays exhibited a resistance phenotype to trimethoprim sulfamethoxazole. Two isolates with *dfrA25* and *dfrA5* cassettes, contrary to our expectation, have not shown resistance to trimethoprim sulfamethoxazole. The reason for this can be found in the presence of weak promoters or the presence of mutations in the region between the two specific sequences of promoters 38.

The *cat* gene that codes for Chloramphenicol Acetyl Transferase (CAT) conferring resistance to chloramphenicol was found in *aadB-cat-blaOXA10-aadA1* cassette array. Although this gene cassette array has already been reported from China 39, identification of this gene cassette array in *K. pnumoniae* isolates was reported for the first time in Iran. Surprisingly, in the current study, this cassette array was found in one chloramphenicol sensitive isolate and despite the presence of *cat* cassette, a large distance between the promoter and the corresponding gene cassette may affect the transcription of *cat* gene in this isolate 38. *blaOXA10* gene found in *aadB-cat-blaOXA10-aadA1* cas-sette array encodes for β-lactamase enzymes conferring resistance to beta-lactam antibiotics such as amoxicillin and ampicillin 40.

## Conclusion

In the present study, the proportional prevalence of integrons and the antibiotic resistance among *K. pneumoniae* isolates clearly shows the importance of integrons in dissemination of antibiotic resistance. *aadA1* was found to be the most prominent gene cassette among 7 different cassette arrays identified in *K. pneumoniae* isolates. Continuous monitoring and characterization of integrons and their associated gene cassettes could be helpful in controlling the rate of antibiotic resistance by planning to take preventive measures to hinder the spread of resistant strains.
